# Analysis of Clinical Profiles and Echocardiographic Cardiac Outcomes in Peripartum Cardiomyopathy (PPCM) vs. PPCM with Co-Existing Hypertensive Pregnancy Disorder (HPD-PPCM) Patients: A Systematic Review and Meta-Analysis

**DOI:** 10.3390/jcm12165303

**Published:** 2023-08-15

**Authors:** Annisa Dewi Nugrahani, Sidik Maulana, Kevin Dominique Tjandraprawira, Dhanny Primantara Johari Santoso, Dani Setiawan, Adhi Pribadi, Amillia Siddiq, Akhmad Yogi Pramatirta, Muhammad Alamsyah Aziz, Setyorini Irianti

**Affiliations:** 1 Department of Obstetrics and Gynecology, Faculty of Medicine, University of Padjadjaran, Dr. Slamet General Hospital Garut, Bandung 45363, West Java, Indonesia; dhannydsog18@gmail.com; 2 Department of Obstetrics and Gynecology, Faculty of Medicine, University of Padjadjaran, Dr. Hasan Sadikin General Hospital, Bandung 45363, West Java, Indonesia; kevin14007@mail.unpad.ac.id (K.D.T.); danisetiawan0323@gmail.com (D.S.); priana1001@gmail.com (A.P.); amel2000id@yahoo.com (A.S.); dryogipramatirta@gmail.com (A.Y.P.); alamsyahaziz9119@gmail.com (M.A.A.); dririanti0901@gmail.com (S.I.); 3 Nursing Internship Program, University of Padjadjaran, Sumedang 45363, West Java, Indonesia; sidik17001@mail.unpad.ac.id

**Keywords:** echocardiography, hypertensive pregnancy disorder, peripartum cardiomyopathy, PPCM

## Abstract

Peripartum cardiomyopathy (PPCM) is a form of new-onset heart failure that has a high rate of maternal morbidity and mortality. This was the first study to systematically investigate and compare clinical factors and echocardiographic findings between women with PPCM and co-incident hypertensive pregnancy disorders (HPD-PPCM) and PPCM-only women. We followed the Preferred Reporting Items for Systematic Review and Meta-Analysis (PRISMA) framework. We used four databases and a single search engine, namely PubMed/Medline, Scopus, Web of Science, and Cochrane. We used Cochrane Risk of Bias (RoB) 2.0 for quality assessment. Databases were searched for relevant articles published from 2013 to the end of April 2023. The meta-analysis used the DerSimonian–Laird random-effects model to analyze the pooled mean difference (MD) and its *p*-value. We included four studies with a total of 64,649 participants and found that systolic blood pressure was significantly more likely to be associated with the PPCM group than the HPD-PPCM group (SMD = −1.63) (95% CI; −4.92,0.28, *p* = 0.01), while the other clinical profiles were not significant. HPD-PPCM was less likely to be associated with LVEF reduction (SMD = −1.55, [CI: −2.89, −0.21], *p* = 0.02). HPD-PPCM was significantly associated with less LV dilation (SMD = 1.81; 95% (CI 0.07–3.01), *p* = 0.04). Moreover, HPD-PPCM was less likely to be associated with relative wall thickness reduction (SMD = 0.70; 95% CI (−1.08–−0.33), *p* = 0.0003). In conclusion, PPCM and HPD-PPCM shared different clinical profiles and remodeling types, which may affect each disease’s response to pharmacological treatment. Patients with HPD-PPCM exhibited less eccentric remodeling and seemed to have a higher chance of recovering their LV ejection fraction, which means they might not benefit as much from ACEi/ARB and beta-blockers. The findings of this study will guide the development of guidelines for women with PPCM and HPD-PPCM from early detection to further management.

## 1. Introduction

Peripartum cardiomyopathy (PPCM) is defined as a form of new-onset heart failure with reduced left ventricular ejection fraction (LVEF) during the peripartum period in the absence of other identified etiologies [1]. In the United States (US), this cardiomyopathy affects 1/1000 to 1/4000 pregnancies. The consequences are devastating for both mothers and infants. PPCM accounts for 5% of heart transplants in women in the United States, as an alternative treatment is not offered in the majority of the world [2]. In underdeveloped countries, up to 25% of mothers with PPCM die within 5 years [3], with neonatal mortality rates ranging from 50% to 75% [4,5].

The etiology of PPCM is enigmatic. There have been numerous hypotheses proposed, including viral myocarditis, autoimmunity, and fetal microchimerism. In 1938, Hull and Hidden [6] discovered a link between postpartum heart failure and hypertensive heart disease when they discovered that >85% of instances of “toxic” postpartum heart disease were related to hypertension, which was double the frequency reported in their control group. PE has frequently been recognized as an independent risk factor for the development of PPCM since the publication of these important studies [7,8,9]. Hypertensive pregnancy disorder (HPD), including preeclampsia, is a significant risk factor for PPCM since women with preeclampsia have a 10–20 times higher risk of PPCM than non-hypertensive controls [6]. While an anti-angiogenic state could theoretically provide a relationship between the two disorders, not all women with HPD have superimposed PPCM [10,11]. Identifying preeclamptic women at high risk of PPCM may lead to focused quality improvement care initiatives, allowing for the early identification of this cardiomyopathy and reducing the burden of unfavorable outcomes associated with late presentation [12]. However, the risk factors for PPCM in HPD women are still not widely known.

Given that less than 20% of women with PPCM have co-incident HPD including preeclampsia, understanding the impact of HPD on PPCM outcomes should assist clinicians in anticipating complications and counseling patients more accurately [13]. Preeclampsia and other HPD-related structural cardiac alterations, including left ventricular concentric remodeling and diastolic dysfunction, may influence the clinical course of women with PPCM. A previous study showed that when PPCM was aggravated with preeclampsia or another HPD (HPD-PPCM group), the pattern of LV remodeling was noticeably different than in PPCM alone. Greater LV dilatation and a decrease in relative LV wall thickness were observed in patients with PPCM only, which was consistent with the traditional “eccentric” LV remodeling. In contrast, neither LV dilation nor a decrease in relative wall thickness were linked with the decline in LV ejection fraction (LVEF) in patients with HPD-PPCM, which was more compatible with a concentric pattern of LV remodeling [3,4,5]. While various small studies with contradictory results have addressed the impact of HPD on PPCM outcomes [7,8,9,12,14,15,16], only two studies reported with sample sizes of less than 40 patients specifically focused on co-incident preeclampsia [14,15].

To the best of our knowledge, there have been no systematic reviews or meta-analyses comparing echocardiographic findings and their associated sociodemographic and clinical factors between women with PPCM and co-incident HPD (HPD-PPCM) and those with PPCM and no HPD (PPCM). It is postulated that PPCM and HPD-PPCM share different types of remodeling, which may affect each disease’s response to pharmacological treatment. A previous study revealed that different types of remodeling have clinical biomarkers and phenotypes that are distinctly different. Moreover, compared to patients with eccentric LV remodeling, people with concentric LV remodeling might not benefit as much from angiotensin-converting enzyme inhibitors, angiotensin receptor blockers, and beta-blockers [7,8,9,14,15,16].

Thus, this study aims to systematically investigate and compare clinical factors and echocardiographic findings between women with PPCM and co-incident HPD (HPD-PPCM) and those with PPCM, which has never been reported before. Since there have been no published guidelines for managing women with PPCM comprehensively, this study intends to guide the development of further provisions and guidelines for women with PPCM worldwide, from early detection to further management, in order to reduce the burden of unfavorable outcomes associated with late presentation of PPCM and PPCM with co-existing HPD including preeclampsia (HPD-PPCM).

## 2. Materials and Methods

### 2.1. Study Design

This study was presented as a systematic review and meta-analysis. The systematic review followed PRISMA and the Cochrane Collaboration’s reporting item for systematic reviews and meta-analyses [17]. PROSPERO registered this study with CRD number (CRD42023428302).

### 2.2. Eligibility Criteria

This systematic review’s inclusion criteria adhered to the PECO (Population, Exposure, Comparison, Outcome) approach. Women with PPCM with or without the co-existence of hypertensive pregnancy disorder (HPD) comprised the study population. The exposure or prognostic factors were risk factors. We extracted information on the following clinical risk factors potentially associated with PPCM and superimposed PPCM among women with HPD (HPD-PPCM), which are: maternal age, gravidity, tobacco use, chronic hypertension history, baseline systolic blood pressure, baseline diastolic blood pressure, and medical therapy initiated after diagnosis (furosemide and beta-blocker). This study compares the risk factors and echocardiographic findings as outcomes in PPCM vs. HPD-PPCM patients. The primary outcome of echocardiographic findings was a composite of cardiovascular indicators that may describe the severity of PPCM and type of remodeling such as left ventricular ejection fraction (LVEF) reduction, left ventricular (LV) dilation, and relative wall thickness reduction.

Studies were included if they were published in peer-reviewed journals, contained original quantitative data, and were written in English. Review and abstract-only articles were also excluded. No restrictions were placed on the publication date of studies. Studies were identified through searching electronic databases, scanning reference lists, and consulting with experts in the field.

### 2.3. Search Strategy and Study Selection

To identify relevant studies for this systematic review and meta-analysis, a comprehensive search of multiple databases was conducted. This search strategy was applied to the PubMed/Medline, Scopus, Web of Science, and Cochrane databases, which were reviewed from their inception up to April 2023. For the purpose of the current review, cases described as preeclampsia, eclampsia, chronic hypertension, gestational hypertension, and chronic hypertension with superimposed preeclampsia were grouped as HPD-PPCM.

The search strategy was developed using a combination of keywords and MeSH terms related to PPCM, preeclampsia (PE), and echocardiographic findings. The search terms used are described in Supplementary File S1. The ‘related articles’ feature and reference lists of included articles were also searched to identify additional relevant studies. Two independent reviewers (ADN and SM) screened all articles for eligibility, and any discrepancies were resolved through discussion with a third reviewer (DPJS). Studies were included if they contained original quantitative data regarding factors and echocardiographic outcomes of PPCM and HPD-PPCM. After the screening had been completed, the reference manager automatically removed duplicates of the article using Mendeley (Mendeley Ltd., New York, NY, USA).

### 2.4. Data Analysis and Quality Assessment

Three authors (ADN, DPJS, and SM) independently extracted data using a standardized data extraction form that included details for risk factors which are maternal age, gravidity, tobacco use, chronic hypertension history, baseline systolic blood pressure, baseline diastolic blood pressure, and medical therapy initiated after diagnosis (furosemide and beta-blocker) as well as echocardiographic findings which are left ventricular ejection fraction (LVEF) reduction, left ventricular (LV) dilation, and relative wall thickness reduction. First, we thoroughly read the studies to identify relevant text related to our research question. Next, we identified recurring themes and categories from the data. We used an iterative process to refine these themes and ensure they accurately represent the data. In cases of disagreement between two authors, a third author (DPJS) served as a mediator.

We evaluated the quality of the non-randomized clinical trials using Cochrane Reviews for Nonrandomized Studies of Intervention (ROBINS-I). Two authors independently evaluated the mentioned studies (ADN and SM). During the evaluation, the following factors were considered: bias resulting from the randomization process, bias resulting from deviation from the intended intervention, bias resulting from missing outcome data, bias in the measurement of the outcome, and bias in the selection of the reported results. Discourse addressed divergent perceptions regarding the quality of the study.

### 2.5. Statistical Analysis

This meta-analysis was performed using RevMan (RevMan International, Inc., New York, NY, USA), Comprehensive Meta-Analysis (CMA) for statistical analysis (Biostat Inc., Englewood, CO, USA), and Jamovie. We calculated pooled effect size estimates as mean differences with 95% confidence intervals using the DerSimonan–Laird method formula (CI). The inconsistency index (I^2^) and subgroup analysis using the Chi-square test were used to assess potential reasons for heterogeneity. An I^2^ of more than 50% and a *p*-value lower than 0.05 were considered significant for heterogeneity [18]. A random-effects model accounted for interstudy variability regardless of study heterogeneity [18,19,20]. We decided to assess statistical significance using a two-tailed *p*-value of 0.05.

## 3. Results

### 3.1. Study Selection

**Figure 1:**
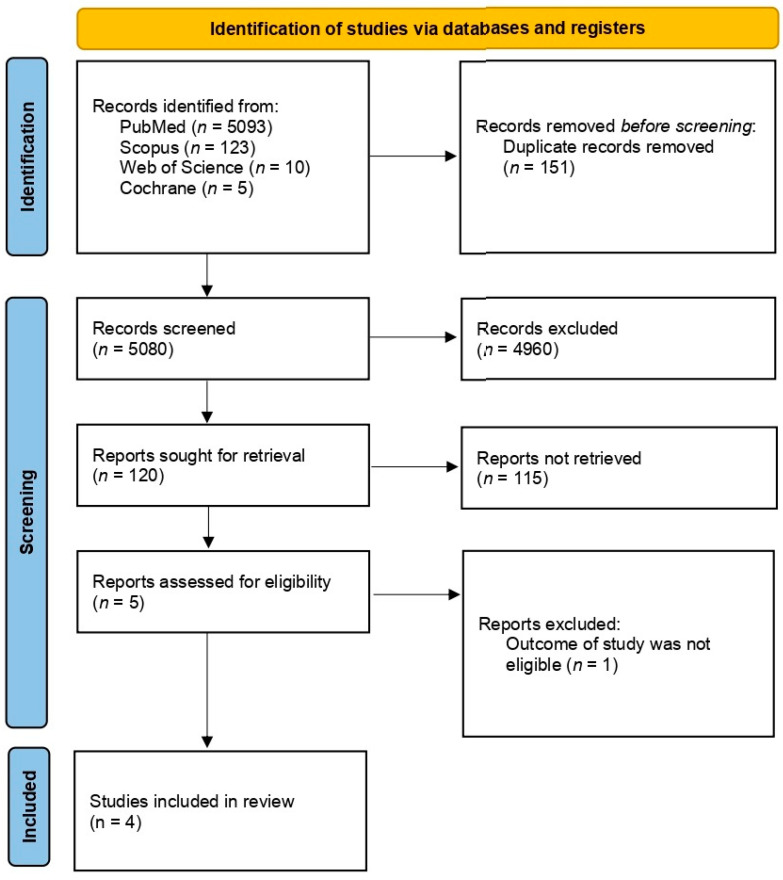
PRISMA flow diagram.

An initial search was conducted in electronic databases, including Scopus, PubMed/Medline, Web of Science, and Cochrane, which yielded a total of 5903 articles. After removing 151 articles due to duplication, the remaining 5080 articles were screened for eligibility. Of those, five studies were evaluated to determine eligibility and a full-text article was excluded from subsequent analysis since it did not present the outcome of interest (this paper separated PPCM with chronic hypertension and PPCM with preeclampsia) [14,15,21,22]. Ultimately, four studies were included in the analysis. Figure 1 illustrates the selection process for identifying the studies included in this review.

### 3.2. Characteristics of Included Studies

The included studies in this systematic review and meta-analysis were similar in terms of study design and country of origin. Among the four studies included, three were retrospective studies, and one a was prospective study. The majority of the studies were conducted in the United States of America (USA) (two studies), in addition to South Africa (one study) and Sweden (one study), indicating that currently, this type of study is rare and is still focusing on developed countries’ populations. This study included a total of 64,649 participants. The age distribution of the study participants ranged from 15 to 55 years old. The patterns of study designs and data included in this review provide a comprehensive understanding of the comparison of sociodemographic factors, clinical factors, and echocardiographic outcomes among PPCM vs. HPD-PPCM patients. There were no significant differences in the characteristics of maternal age at diagnosis and gravidity between the PPCM and HPD-PPCM groups in all the included studies (Table 1).

### 3.3. Risk of Bias

**Figure 2:**
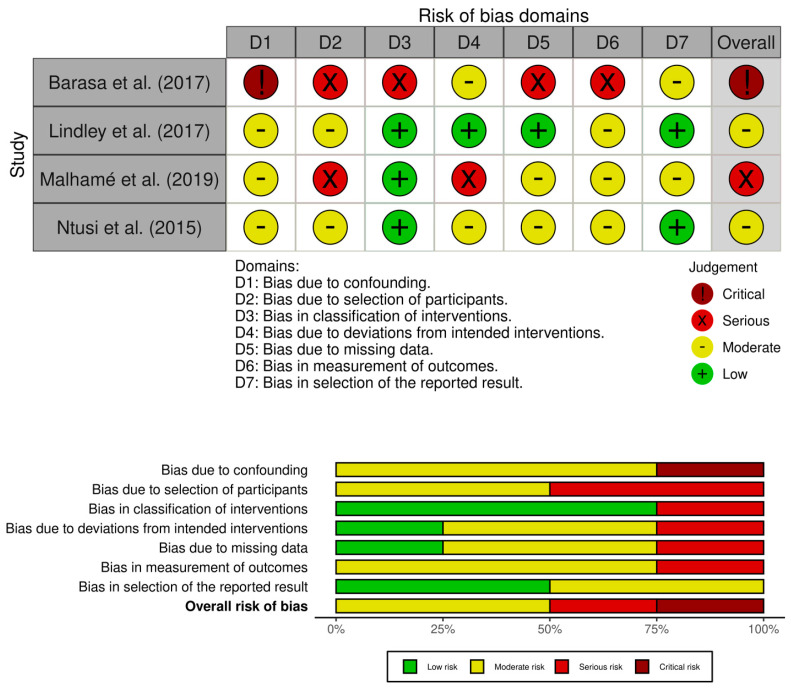
Risk of bias assessment with ROBINS-I [14,15,21,22].

Based on the risk of bias assessment using ROBINS-I, there are two studies with moderate risk, one study with serious risk, and one study with a critical risk of bias. The risk of bias domain with moderate level was dominated by D1 (bias due to confounding), D2 (bias due to selection of participants), D6 (bias in measurements of outcomes), and D7 (bias in reported results). The serious risk bias was due to D2 (bias due to the selection of participants) and D4 (bias due to deviations from intended interventions). The critical risk of bias study was due to critical D2 (bias due to selection of participants) Moreover, the risk of bias was dominated by D1 (bias due to confounding) and D6 (bias in measurement of outcomes) (see Figure 2).

### 3.4. Study Outcomes

#### 3.4.1. Clinical Profiles

##### Chronic Hypertension

**Figure 3:**
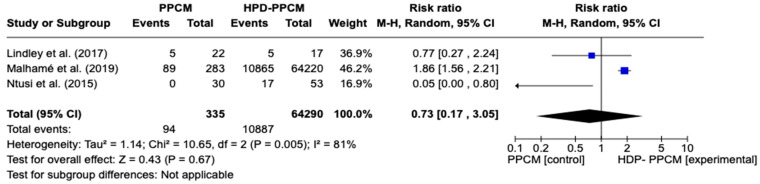
Forest plot of the impact of chronic hypertension on PPCM vs. HPD-PPCM [15,21,22].

Similar to tobacco use analysis, three studies (Lindley et al., Ntusi et al., and Malhame et al.) [15,21,22] included an analysis of baseline history of chronic hypertension in their studies. Figure 3 reveals that chronic hypertension was more likely to influence the PPCM group rather than the HPD-PPCM group (RR = 0.73) [95% CI; 0.17,3.05, *p* = 0.67], which may favor diseases’ outcomes, although not significant. A high heterogeneity was also identified (I^2^ = 81%, *p* = 0.005).

##### Systolic Blood Pressure (SBP)

**Figure 4:**
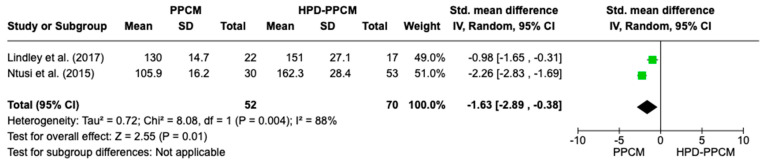
Forest plot thehe impact of systolic blood pressure on PPCM vs. HPD-PPCM [15,22].

Only two studies (Lindley et al. and Ntusi et al.) [15,22] included an analysis of baseline systolic blood pressure in their studies. Figure 4 revealed that systolic blood pressure was significantly more likely to less influence the PPCM group rather than the HPD-PPCM group (SMD = −1.63) [95% CI; −4.92,0.28, *p* = 0.01], which may favor diseases’ outcome significantly. A high heterogeneity was also identified (I^2^ = 88%, *p* = 0.004).

##### Diastolic Blood Pressure

**Figure 5:**
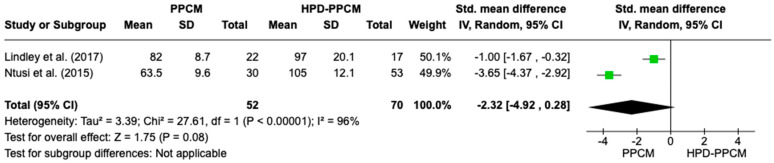
Forest plot of the impact of diastolic blood pressure on PPCM vs. HPD-PPCM [15,22].

Only two studies (Lindley et al. and Ntusi et al.) [15,22] included an analysis of baseline diastolic blood pressure in their studies. Figure 5 revealed that diastolic blood pressure was less likely to influence the PPCM group rather than the HPD-PPCM group with (SMD = −2.32) [95% CI; −4.92,0.28, *p* = 0.08], which may favor diseases’ outcome, although not significant. A high heterogeneity was identified (I^2^ = 96%, *p* = <0.00001).

##### Medical Therapy Initiated after Diagnosis (Furosemide)

**Figure 6:**
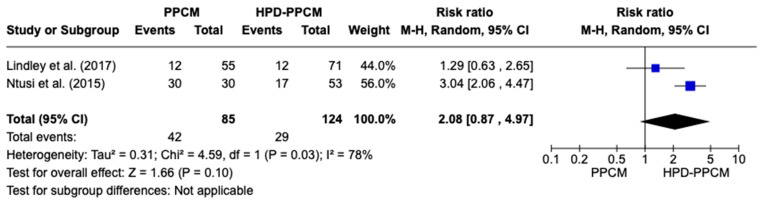
Forest plot of the impact of furosemide on PPCM vs. HPD-PPCM initiated after diagnosis [15,22].

Only Lindley et al. and Ntusi et al. [15,22] included an analysis of the initiation of furosemide in their studies. Although not significant, Figure 6 revealed that furosemide was more likely used in HPD-PPCM patients after diagnosis (RR = 2.08) [95% CI; 0.71,8.34, *p* = 0.10], which may favor the disease’s outcome. A high heterogeneity was identified (I^2^ = 78%, *p* = 0.03).

##### Medical Therapy Initiated after Diagnosis (Beta-Blocker)

**Figure 7:**
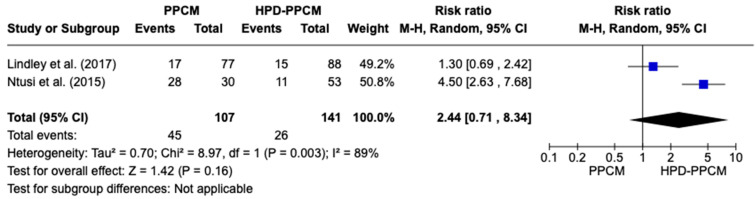
Forest plot of the impact of beta blocker on PPCM vs. HPD-PPCM Initiated after Diagnosis [15,22].

Similar to previous analyses of furosemide initiation after diagnosis, only two studies (Lindley et al. and Ntusi et al.) [15,22] included an analysis of the initiation of beta-blockers in their studies. Figure 7 revealed that beta-blocker was more likely used in HPD-PPCM patients after diagnosis, although not significant (RR = 2.44) [95% CI; 0.71,8.34, *p* = 0.16], which may favor diseases’ outcomes. A high heterogeneity was identified (I^2^ = 89%, *p* = 0.003).

#### 3.4.2. Echocardiographic Findings

##### The Impact of PPCM vs. HPD-PPCM on LVEF Reduction

**Figure 8:**
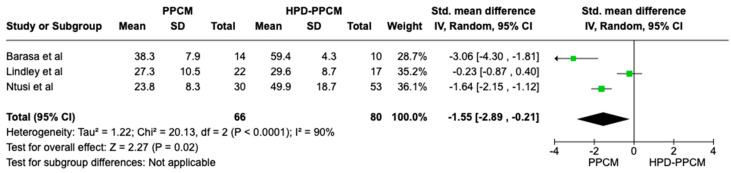
Forest plot of the impact of PPCM vs. HPD-PPCM on LVEF reduction [14,15,22].

In the LVEF outcome analysis consisting of all studies included, HPD-PPCM was less likely to have LVEF reduction compared to the PPCM group with SMD = −1.55 [95% CI: −2.89, −0.21] (Figure 8), and it was considered significant (*p* = 0.02). Moreover, a high heterogeneity was identified (I^2^ = 90%, *p* < 0.00001).

##### The Impact of PPCM vs. HPD-PPCM on LV Dilation

**Figure 9:**
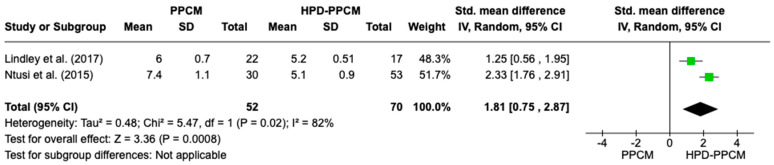
Forest plot of the impact of PPCM vs. HPD-PPCM on LV dilation [15,22].

Two studies were included in the analysis of LV dilation. Lindley et al. [15] showed that HPD-PPCM only was not associated with LV dilation; meanwhile, Ntusi et al. [22] showed that HPD-PPCM was related to greater LV dilation. Figure 9 is a forest plot depicting that HPD-PPCM was significantly associated with less LV dilation, with SMD = 1.81; 95% CI (0.07–3.01)] and *p* = 0.04. However, a high heterogeneity was identified (I^2^ = 82%, *p* = 0.02).

##### The Impact of PPCM vs. HPD-PPCM on Relative Wall Thickness Reduction

**Figure 10:**
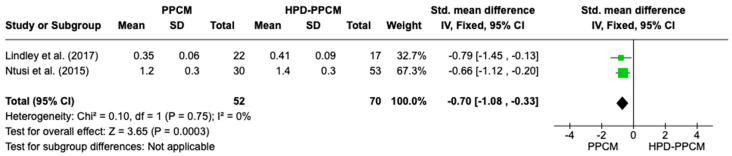
Forest plot of the impact of PPCM vs. HPD-PPCM on relative wall thickness reduction [15,22].

Both Lindley et al. and Ntusi et al. [15,22] showed that HPD-PPCM patients were less likely to develop a reduction in relative wall thickness. Figure 10 is a forest plot depicting that HPD-PPCM was less likely and the PPCM group was more likely to have a relative wall thickness reduction [SMD = 0.70; 95% CI (−1.08–−0.33)], with *p* = 0.0003. No heterogeneity was identified (I^2^ = 0%, *p* = 0.0003).

## 4. Discussion

### 4.1. Principal Findings

PPCM is a new onset of heart failure that has a high rate of maternal morbidity and mortality [1,2,3,4,5]. Preeclampsia and other HPD-related structural cardiac alterations, including left ventricular concentric remodeling and diastolic dysfunction, may influence the clinical course of women with PPCM. We focused on investigating the comparison of echocardiographic findings and their associated sociodemographic and clinical factors between women with PPCM and co-incident HPD (HPD-PPCM) and those with PPCM and no HPD (PPCM). This study revealed that systolic blood pressure was significantly more likely to influence the PPCM group rather than the HPD-PPCM group (SMD = −1.63) [95% CI; −4.92, 0.28, *p* = 0.01], which may favor diseases’ outcome significantly, while the other sociodemographic or clinical profiles were not considered significant. To illustrate risk factors for HPD-PPCM vs. PPCM’s course of the disease, our study reported that HPD-PPCM was associated with older or more advanced maternal age, a greater number of gravidity, tobacco use, and chronic hypertension rather than the PPCM-only group, although they were not significant. Medical therapy such as furosemide and beta-blockers were more likely used in HPD-PPCM patients after diagnosis and might favor the disease’s course, although not significant.

Hypertensive pregnancy disorder was found in 37% of women with PPCM, while PPCM was found in 22% of these women, compared to an average worldwide background rate of 5% [2,23]. Preeclampsia is a common hypertensive pregnancy disease that has been linked to short-term and long-term cardiovascular dysfunction-related postpartum morbidity and mortality in both [12,23,24,25,26,27,28]. Preeclampsia amplifies the systemic angiogenic imbalance that develops in PPCM [29]. Soluble FLT1 (sFLT1) is one of the VEGF inhibitors secreted by the placenta in human beings, resulting in angiogenic imbalance and high blood pressure as one of its manifestations [29]. Although sFLT1 levels are higher in patients with preeclampsia than in controls, they are higher in women with PPCM [29]. After delivery, sFLT1 levels rapidly decrease. Even in the absence of pregnancy, exogenous sFlt1 was sufficient to induce severe systolic dysfunction in an in vivo study. Furthermore, preeclampsia patients have significantly higher sFlt1 levels, which is probably considered a high-risk factor for PPCM [15]. These findings are in line with our study, which revealed that systolic and diastolic blood pressure is less likely to influence the PPCM group rather than HPD-PPCM due to the basic nature of each disease, and HPD-PPCM patients tend to have a higher mean baseline blood pressure.

Based on echocardiology findings, HPD-PPCM was significantly less likely to have LVEF reduction compared to the PPCM group, with SMD −0.67 [95% CI: −3.04, 1.71] (*p* = 0.02). HPD-PPCM was significantly associated with less LV dilation, with SMD = 1.81; 95% CI (0.07–3.01)] and *p* = 0.04. Moreover, HPD-PPCM was less likely, and the PPCM group was more likely to have a relative wall thickness reduction [SMD = 0.70; 95% CI (−1.08–−0.33)], with *p* = 0.0003. PPCM and HPD-PPCM share different types of remodeling, which may affect each disease’s response to pharmacological treatment. These findings suggest that in PPCM patients with HPD, the patterns of LV remodeling and LV function recovery were noticeably different and considered to share different pathophysiology mechanisms. Patients with HPD-PPCM exhibited less eccentric remodeling (more concentric) and seemed to have a higher chance of recovering their LV ejection fraction.

Although it has been demonstrated that decreases in radial, circumferential, and longitudinal strain occur before an ejection fraction loss, preeclampsia and HPD are associated with afterload-driven left ventricular concentric remodeling and impaired diastolic function [12,15,22]. The left ventricle is more likely to be affected by the rise in afterload that comes with hypertension than the right ventricle. However, HPD-PPCM patients seem to have a higher chance of recovering their LV ejection fraction, as mentioned, which might be due to more aggressive optimization of heart failure therapies in concomitant HPD-PPCM circumstances rather than PPCM-only patients.

When hypertension is resolved (with the removal of an increased afterload), recovery of LV function and reverse remodeling in HPD-PPCM may occur more in individuals with dilated cardiomyopathy than from other causes. Reverse remodeling has been seen in various series of PPCM-affected women up to 2–5 years after diagnosis [24,30]. Based on an earlier study, HPD-PPCM patients were more likely to exhibit symptoms of ‘conventional’ dilated cardiomyopathies, including more severe biventricular dysfunction, frequent electrocardiographic changes like left bundle branch block, and a higher frequency of family history of cardiomyopathy [31,32].

Moreover, a previous study revealed that different types of remodeling have clinical biomarkers and phenotypes that are distinctly different. In PPCM, a previous study found that compared to two control groups of women with prior severe preeclampsia and prior uncomplicated pregnancies, respectively, who were matched on age and year of index delivery, women with PPCM had significantly higher levels of sFlt-1, PlGF, copeptin, and NT-proBNP and more frequently detectable cathepsin D (CD) activity. However, prior systematic reviews on PPCM diagnosis using biomarkers and echocardiography have demonstrated that no parameter has consistently performed well across all investigations. In numerous trials, echocardiographic parameters—including strain profiles and biomarkers—proved important in predicting the prognosis of patients with PPCM [33,34]. We propose that further investigation would be needed to evaluate the association between the predictive value of biomarkers, genetics, polymorphism, and PPCM vs. HPD-PPCM. Moreover, the genetic foundations of PPCM are still poorly understood. In addition, a study conducted by Goli et al. in 2021 [35] showed some genetic overlap between PPCM and dilated cardiomyopathy, indicating that PPCM may benefit from gene-specific therapy strategies being explored for dilated cardiomyopathy. A total of 10% of women with PPCM had TTN variations that are truncating (TTNtvs). There were no appreciable differences in the timing of presentation after delivery, the prevalence of preeclampsia, or the rates of clinical recovery. A case of PPCM with predominately diastolic dysfunction was reported by Ballo et al. [36] and was treated with bromocriptine to inhibit prolactin. Through removing the cleaved form of prolactin despite activating the cleaving enzyme, bromocriptine, a dopamine agonist that decreases prolactin production, may improve outcomes in patients with peripartum cardiomyopathy. The study also discovered that bromocriptine therapy reduced the development of PPCM in animals lacking STAT3 and improved the cardiac output function in women with PPCM. Bromocriptine prevents prolactin production from the pituitary, which suppresses lactation. However, its utilization in acute PPCM is not linked to any substantial adverse effects, including no thromboembolism occurrences. Additional research is required to report the clinical outcomes of newborns whose mothers use this treatment [37]. However, none of our included studies compared any biomarkers, genetics, and polymorphisms, as well as bromocriptine utilization in PPCM vs. HPD-PPCM.

When patients are successfully up-titrated, the point estimates of the hazard ratio are lower in eccentric hypertrophy than in concentric hypertrophy. The Valsartan Heart Failure Trial (Val-HeFT) study revealed that valsartan significantly reduced relative and absolute risk in patients with the largest LV internal diastolic dimensions [32]. A previous study also postulated that in addition to ejection percentage, the shape of the left ventricle may also have an impact on how well beta-blockers and angiotensin-converting enzyme inhibitor (ACEi)/angiotensin receptor blockers (ARBs) respond to up-titration. Moreover, compared to patients with eccentric LV remodeling, people with concentric LV remodeling (HPD-PPCM) might not benefit as much from angiotensin-converting enzyme inhibitors, angiotensin receptor blockers, and beta-blockers [7,8,9,14,15,16].

### 4.2. The Implication for Clinical Practice

Identifying preeclamptic women at high risk of PPCM may lead to focused quality improvement care initiatives. This condition leads to the early identification of this cardiomyopathy and reduces the burden of unfavorable outcomes associated with late presentation. Moreover, this meta-analysis study also gives insight. PPCM and HPD-PPCM share different types of remodeling, which may affect each disease’s response to pharmacological treatment. Compared to patients with eccentric LV remodeling, people with concentric LV remodeling might not benefit as much from angiotensin-converting enzyme inhibitors, angiotensin receptor blockers, and beta-blockers. The findings in this study will guide the development of guidelines for women with PPCM worldwide, from early detection to further management, in order to lessen the burden of unfavorable outcomes associated with the late presentation of PPCM and HPD-PPCM.

### 4.3. Strength and Limitations

To the best of our knowledge, this is the first systematic review or meta-analysis comparing clinical factors and echocardiographic findings between women with PPCM and co-incident HPD (HPD-PPCM) and those with PPCM and no HPD (PPCM); that is the primary strength of this study. This study also uses a careful assessment of the reviewed studies’ risk of bias. However, several limitations should be acknowledged. First, after bias was assessed, the results showed that most studies have a moderate risk of bias, with one study having a serious risk of bias and the other study having a critical risk of bias. The risk of bias domain with moderate level was dominated by D1 (bias due to confounding), D2 (bias due to selection of participants), D6 (bias in measurements of outcomes), and D7 (bias in reported results). Moreover, the risk of bias was dominated by D1 (bias due to confounding) and D6 (bias in measurement of outcomes). Thus, it has quite an effect on the overall biased results. Second, the study was limited to be performed in USA and Africa. Therefore, this study could not be generalized to other countries. Third, this study was limited to the number of included studies due to a lack of studies that reported the comparison of PPCM vs. HPD-PPCM clinical profiles and echocardiographic outcomes. Not all studies reported clinical profiles, such as medical therapy initiated after diagnosis. Only a few studies reported the use of ACEi/ARBs. Thus, only furosemide and beta-blocker use were reported by related studies. Therefore, only a few outcomes can be performed with meta-analysis.

## 5. Conclusions

PPCM and HPD-PPCM share different clinical profiles as well as types of remodeling, which may affect each disease’s response to pharmacological treatment. Systolic blood pressure was significantly more likely associated with the PPCM group than the HPD-PPCM group, while the other clinical profiles were not significant. HPD-PPCM was less likely to have LVEF reduction, less LV dilation, and was less likely to have a relative wall thickness reduction. In PPCM patients with HPD, the patterns of LV remodeling and LV function recovery were noticeably different and considered to share different pathophysiology mechanisms. Patients with HPD-PPCM exhibited less eccentric remodeling (more concentric) and seemed to have a higher chance of recovering their LV ejection fraction, which might not benefit as much with angiotensin-converting enzyme inhibitors, angiotensin receptor blockers, and beta-blockers. The findings in this study will guide the development of guidelines for women with PPCM worldwide, from early detection to further management, in order to lessen the burden of unfavorable outcomes associated with the late presentation of PPCM and HPD-PPCM. However, further studies are needed to emphasize the intricate connection between PPCM and HPD, as well as meta-analysis regarding the clinical outcomes of PPCM after follow-up.

## Figures and Tables

**Table 1:** Characteristics of included studies (*n* = 4 studies).

Study	PPCM		HPD-PPCM		*p*-Value
	Mean (SD)	N	Mean (SD)	N	
**Design**					
Barasa et al. (2017) [14]	Retrospective cohort				
Lindley et al. (2017) [15]	Retrospective cohort				
Ntusi et al. (2015) [22]	Retrospective cohort				
Malhamé et al. (2019) [21]	Prospective cohort				
Sociodemographic Profiles					
**Location**					
Barasa et al. (2017) [14]	Sweden				
Lindley et al. (2017) [15]	USA				
Ntusi et al. (2015) [22]	USA				
Malhamé et al. (2019) [21]	South Africa				
**Maternal Age at Diagnosis**					
Lindley et al. (2017) [15]	29.30 (5.090)	22	27.40 (7.40)	17	0.90
Ntusi et al. (2015) [22]	31.50 (7.50)	30	29.60 (6.60)	53	
Malhamé et al. (2019) [21]	30.20 (5.80)	64,220	31.80 (6.80)	283	
**Gravidity**					
Lindley et al. (2017) [15]	3.10 (1.90)	22	2.60 (2.20)	17	0.13
Ntusi et al. (2015) [22]	2.40 (0.70)	30	2.20 (0.60)	53	
**Tobacco Use**	(Even, Total)		(Event, Total)		
Lindley et al. (2017) [15]	4	22	4	17	0.10
Malhamé et al. (2019) [21]	8	283	1143	64,220	
Ntusi et al. (2015) [22]	7	30	5	53	

Note: PPCM (peripartum cardiomyopathy); HPD-PPCM (peripartum cardiomyopathy with co-existing hypertensive pregnancy disorder comorbidities); USA (United States of America); SD (standard deviation). *p*-value was created through meta-analysis.

## Data Availability

More data are available from the author. Please contact the corresponding author for more data.

## Supplementary Materials

The following supporting information can be downloaded at: https://www.mdpi.com/article/10.3390/jcm12165303/s1, File S1: Searching strategy.
