# Supplementary material for: Analysis of Clinical Profiles and Echocardiographic Cardiac Outcomes in Peripartum Cardiomyopathy (PPCM) vs. PPCM with Co-Existing Hypertensive Pregnancy Disorder (HPD-PPCM) Patients: A Systematic Review and Meta-Analysis
Source: J Clin Med. 2023 Aug 15;12(16):5303. doi: 10.3390/jcm12165303 (PMC10455411; doi:10.3390/jcm12165303)
Supplement: Supplementary file 1 [file jcm-12-05303-s001.zip › jcm-2464521-SI.pdf]

Supplementary File S1. Searching strategy

#1 cardiomyopathie* OR “heart failure” OR “peripartum cardiomyopathy*” OR PPCM

#2 preeclampsia OR pre-eclampsia OR PE OR "edema-proteinuria-hypertension" OR EPH OR "gestational edema-proteinuria-hypertension" OR GEPH OR "proteinuria edema hypertension gestosis"

#3 “peripartum period” OR peripartum OR pregnanc*

#4 echocardiography OR echocardiography outcome

#5 clinical outcome* OR “patient outcome*” OR “health outcome*” OR prognosis
